# Effects of a Calorie-Restricted Cafeteria Diet and Oleuropein Supplementation on Adiposity and mRNA Expression of Energy Balance Related Genes in Obese Male Rats

**DOI:** 10.3390/metabo13020147

**Published:** 2023-01-18

**Authors:** Alex Subias-Gusils, Adam Álvarez-Monell, Noemi Boqué, Antoni Caimari, Roger Mariné-Casadó, Rosa M. Escorihuela, Montserrat Solanas

**Affiliations:** 1 Institut de Neurociències, Universitat Autònoma de Barcelona, 08193 Bellaterra, Spain; 2 Departament de Psiquiatria i Medicina Legal, Facultat de Medicina, Universitat Autònoma de Barcelona, 08193 Bellaterra, Spain; 3 Department of Cell Biology, Physiology and Immunology, Faculty of Medicine, Universitat Autònoma de Barcelona, 08193 Bellaterra, Spain; 4 Eurecat, Centre Tecnològic de Catalunya, Biotechnology Area and Technological Unit of Nutrition and Health, 43204 Reus, Spain

**Keywords:** adiponectin, calorie restriction, dietary supplements, hypothalamus, leptin, metabolic syndrome, white adipose tissue

## Abstract

Supplementation with natural bioactive compounds has been proposed to be a complementary tool to the calorie-restricted diets and physical exercise programs used to tackle human overweight, obesity and Metabolic syndrome. Herein, we evaluated the effects of 14 weeks of calorie-restricted cafeteria diet either alone or combined with oral administration of the polyphenol oleuropein in obese adult male rats, compared with a control group fed standard chow and a group fed cafeteria diet. Animals were sacrificed at the age of 26 weeks and several tissues of interest were removed. The results showed that both dietary interventions reduced the adiposity index (*p* < 0.05 and *p* < 0.01, respectively), and specifically the abdominal fat depots (mesenteric: *p* < 0.01 and *p* < 0.01, respectively; and epididymal: both diets *p* < 0.001) and restored the decreased soleus skeletal muscle mass. Both interventions decreased leptin mRNA expression in mesenteric white adipose tissue (*p* < 0.05) and normalized hypothalamic *Agrp* mRNA expression compared to cafeteria-fed obese rats (*p* < 0.05). However, only the calorie-restricted cafeteria diet supplemented with oleuropein induced additional lower retroperitoneal adipose accretion (*p* < 0.05) and increased hypothalamic leptin receptor mRNA levels (*p* < 0.05). Experiments with female animals, at different doses and longer intervention periods, are needed to better determine the potential benefits of this dietary treatment.

## 1. Introduction

Obesity is a chronic metabolic disease characterized by an increase of body fat stores due to a chronic positive energy balance [1]. It is associated with numerous comorbidities such as cardiovascular disease, hypertension, type 2 diabetes and certain cancers [2,3]. The acute increase in the prevalence of obesity reported during the recent decades may be associated with the increasing presence of energy dense foods, reduced physical activity and the sedentary lifestyle mainly in developed countries [4].

The hypothalamus (HPT) plays a key role in the regulation of food intake and energy balance. In the arcuate nucleus region, anorexigenic (POMC/CART) and orexigenic (AGRP/NPY) neuron populations activate neuronal pathways that suppress or increase food intake, respectively [5]. Leptin from adipocytes and insulin from the pancreas are implicated in this regulation, acting as peripheral signals of energy status to the central nervous system where they inhibit the orexigenic pathways and stimulate the anorexigenic pathways via their corresponding receptors in these hypothalamic neurons [6]. Therefore, by expressing adipocytokines such as leptin or adiponectin, adipose tissue plays a key role in the regulatory cascades involved in obesity and Metabolic Syndrome (MetS) and in the cross-talk between major organs (brain-HPT, liver, muscles) [7]. Leptin acts in a homeostatic loop that regulates body-fat mass and energy balance not only at the central nervous system level, but also directly at the adipocyte level, regulating lipolysis [8]. Interestingly, obesity is associated with selective leptin resistance, which can lead to increased hyperphagia and rapid weight gain [9].

Dietary supplementation with natural bioactive compounds such as polyphenols, combined with calorie-restricted diets and/or physical exercise programs, has emerged as a promising therapeutic tool in the prevention and management of obesity and metabolic disease. Polyphenols have been shown to modulate energy metabolism by stimulating β-oxidation, inhibiting adipocyte differentiation and inflammation, or counteracting oxidative stress [10]. However, the results obtained in meta-analyses of randomized clinical studies are not consistent and more clinical studies with larger numbers of participants, longer times of exposure and different doses must be conducted to validate them as a realistic strategy against obesity [1,11].

One of the polyphenols that might exhibit potential beneficial effects on obesity-related parameters is the secoiridoid oleuropein (OLE), present in olive (*Olea europaea*) fruits and virgin oils. It is one of the main components of the Mediterranean diet, which is considered the healthiest dietary pattern available to prevent several non-communicable diseases [12]. In experimental animal studies, OLE supplementation was able to reduce dyslipidaemia and improve obesity and diabetes-related alterations such as abdominal fat gain, insulin sensitivity, adipose tissue inflammation and dysbiosis in gut microbiota through its inhibition of the expression of genes related to adipogenesis, and reduction of pro-inflammatory adipokines, among other actions [13,14,15]. These reported effects of OLE are closely associated with its bioavailability and metabolism once it is absorbed in the gastrointestinal tract. Its bioavailability is influenced by the route of administration, interaction with foods and by the extraction processes [16]. OLE plasma concentrations were observed to peak 20 min after oral ingestion in rodents. In humans, the maximum OLE concentration in plasma was observed after 23–93 min and the peak appeared 64 min after ingestion, on average. In rodents, OLE and its metabolic derivates, such as hydroxytyrosol or elenoic acid, were detected in the liver and kidneys. Regarding its excretion, OLE was detected in urine both in rodents and humans, whereas none was detected in the cecum content or faeces, indicating that it may be mainly excreted via urine [17].

Rodent models, specifically rats and mice, are the most widely used preclinical animal models for the study of metabolic disorders such as obesity or type 2 diabetes [18]. We can distinguish two types of animal models depending on the strategy used to induce obesity in the animals: (i) The diet-induced obesity (DIO) models, which use obesogenic diets such as the high-fat (HFD) or cafeteria (CAF) diets; and (ii) the genetic models, which feature the mutation of one (monogenic) or various genes (polygenic) such as the *ob*/*ob* and the C57BL6/J mice, respectively [18,19,20]. One of the DIO models that is most used to investigate obesity disorders and preclinical treatment strategies is the CAF diet, an experimental obesogenic diet based on the administration of highly energetic and palatable human food items, such as biscuits, cheese, muffins and sugared milk, which induces hyperphagia and increases energy intake [21,22,23,24]. In comparison with high-fat/high-sugar diets, it is considered a more robust DIO model in rodents because it better mimics the hedonic hyperphagia observed in obese humans and promotes more severe symptoms [18,25].

In a previous study, we characterized an experimental restricted cafeteria (CAF-R) diet consisting of standard rodent chow supplemented with small portions of CAF items to achieve a 30% calorie-reduction in relation to the total calorie intake of the CAF-fed group [26]. The addition of OLE to the CAF-R diet diminished the hedonic responses to high-sucrose solutions in obese male animals, while preserving the reductions in body weight gain, body mass index (BMI), and daily food and energy intakes; and the improvement of several biochemical parameters associated with obesity [27].

The current study aimed to evaluate whether those dietary interventions changed the mass of adipose depots and skeletal muscles, as well as to characterize the mRNA expression levels of genes involved in the above mentioned hypothalamic anorexigenic and orexigenic pathways and the leptin, leptin receptor and adiponectin mRNA expression levels in white adipose tissue.

## 2. Materials and Methods

### 2.1. Experimental Design

The tissues and samples used in the present study were obtained from the cohort of male Sprague-Dawley rats used in Subias-Gusils et al. [27]. Briefly, two dietary interventions based on the CAF-R diet alone or supplemented with OLE (CAF-RO diet) at a dose of 25 mg/kg·day of an *Olea europaea* leaf extract (Benolea^®^, Frutarom Health BU, Wädenswil, Switzerland) were administered for 14 weeks to CAF diet-induced obese animals. The CAF and CAF-R diets were composed of the following items: bacon, biscuit with pâté, biscuit with cheese, muffins, carrots, jellied sugared milk and standard chow. The amount of each cafeteria food item administered to the CAF-R and CAF-RO animals was readjusted every week based on a 30% reduction in calories relative to the energy consumed by the CAF group. A control (STD) group was fed standard chow ad libitum during the entire experiment [27]. The CAF diet was provided ad libitum to the animals after weaning, starting at the age of 24 days old and continuing for 8 weeks. When the animals were 80 days-old, the obese CAF-fed animals were subdivided into three groups CAF, CAF-R and CAF-RO according to the diet administered until the end of the experiment. 

The average daily intake of macronutrients during the dietary treatments, expressed as the percentage of the average total daily energy intake in kilocalories coming from each macronutrient type, as well as the feed efficiency, are indicated in Table S1. 

The animals were sacrificed at an age of 26 weeks, and the body weights were: STD, 465.9 ± 8.8; CAF, 564.9 ± 15.4; CAF-R, 537.5 ± 15.9; and CAF-RO, 536.6 ± 12.0 (mean ± SEM, grams). Euthanasia was carried out by decapitation after 8 h of fasting, which started in the morning at 6 a.m., leaving 20 min between animals, and sacrifices were performed sequentially between 2 p.m. and 5 p.m. for a total of 3 days. The following tissues were removed: HPT, WAT depots (retroperitoneal -rWAT-, mesenteric -mWAT-, epididymal -eWAT- and inguinal -iWAT-), interscapular brown adipose tissue (iBAT), liver, cecum, gastrocnemius and soleus muscles. Once removed, they were weighed, frozen in liquid nitrogen and stored at −80 °C until further analysis. A macroscopic examination of all tissues was performed before storage. The tissue exeresis was carried out by the same qualified experimenters through the procedure to decrease variability.

The adiposity index was determined as the sum of the eWAT, iWAT, mWAT and rWAT weights (in grams) and expressed as a percentage of body weight (g/kg·100). Abdominal WAT referred to the sum of rWAT, mWAT and eWAT weights, while subcutaneous WAT was considered as the iWAT.

The experimental protocol was approved by the Generalitat de Catalunya (DAAM 9978), following the ‘Principles of laboratory animal care’ and was carried out in accordance with the EU Directive 2010/63/EU for animal experiments and following ARRIVE guidelines.

### 2.2. mRNA Expression Analyses

The entire HPT and 80 mg of mWAT were each homogenized in 1 mL of QIAzol^®^ Lysis Reagent (Qiagen, Barcelona, Spain) using a tissue homogenizer (Polytron™ PT 2100, KINEMATICA^®^) according to the corresponding manufacturers’ protocol (RNeasy Lipid Tissue Mini Kit—Qiagen) to obtain the total RNA from each sample. Quantification and quality (260/280 and 260/230 contamination ratios) of the RNA obtained were analyzed using a NanoDrop™ 1000 Spectrophotometer (Thermo Fisher Scientific, Madrid, Spain). RNA integrity was checked by agarose gel electrophoresis. The cDNA was synthesized from 2 µg/mL of total RNA using the High-Capacity cDNA Reverse Transcription Kit (Applied Biosystems, Madrid, Spain) and the following RT-PCR program: 25 °C for 10 min, 37 °C for 120 min, 85 °C for 5 min and stored at 4 °C. The resulting cDNA was amplified by real-time PCR with TaqMan^®^ methodology in the iCycler MyiQTM Single Color Real-Time PCR Detection System (Bio-Rad Laboratories, Madrid, Spain). Specific Gene TaqMan Assays for *Lepr*, *Insr*, *Pomc*, *Cartpt*, *Npy*, *Agrp*, *Lep*, *Adipoq*, and *Hprt* as the housekeeping control gene, were obtained from Applied Biosystems. The Real-Time PCR program was set as follows: 95 °C for 10 min, 40 cycles of 95 °C for 15 s and 60 °C for 1 min. Each PCR was performed in duplicate. The relative expression of each mRNA was calculated using the 2^−ΔΔCt^ method [28].

The TaqMan^®^ Gene Expression Assays used to amplify the different genes were: Rn01433205_m1 (*Lepr*); Rn00690703_m1 (*Insr*); Rn00595020_m1 (*Pomc*); Rn00567382_m1 (*Cartpt*); Rn00561681_m1 (*Npy*); Rn01431702_g1 (*Agrp*); Rn01433205_m1 (*Lepr*); Rn00565158_m1 (*Lep*); Rn00595250_m1 (*Adipoq*); and Rn01527840_m1 (*Hprt1*).

### 2.3. Statistical Analyses

Statistical analyses were performed using SPSS Statistics 22 (SPSS, Inc., Chicago, IL, USA). The homoscedasticity among groups was measured using Levene’s test. Grubbs’ test was used to determine significant outlier values, and consequently, discarded them for the analyses. One-way ANOVAs followed by Duncan post-hoc test, or Student’s *t* test, were performed to evaluate differences among groups. All the results were expressed as mean ± SEM. The level of statistical significance was set at bilateral 5%.

## 3. Results

### 3.1. Adipose Depots and Other Tissues

**Figure 1:**
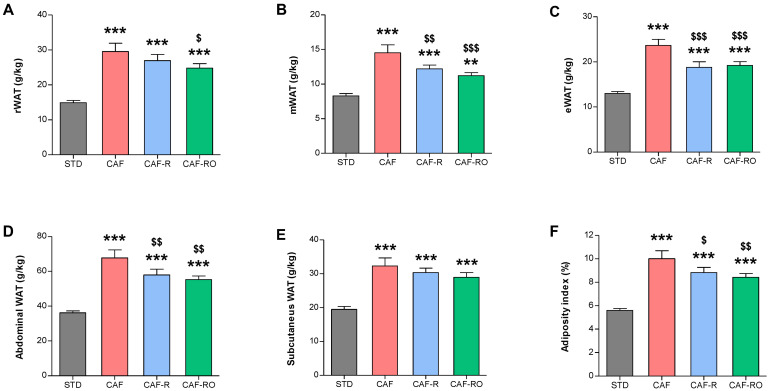
Effects of the dietary treatments on the relative weight of the retroperitoneal WAT (**A**), mesenteric WAT (**B**), epididymal WAT (**C**), abdominal WAT (**D**) and subcutaneous WAT (**E**), and on the adiposity index (**F**). Tissue weights in grams were relativized to the total body weight in kg. Data are expressed as the mean ± SEM. ** *p* < 0.01, *** *p* < 0.001 vs. STD group; $ *p* < 0.05, $$ *p* < 0.01, $$$ *p* < 0.001 vs. CAF group.

The CAF group showed the highest values of mWAT, eWAT, abdominal WAT and adiposity index; the CAF-R and CAF-RO groups displayed intermediate values; and the STD group the lowest ones (*p* < 0.001; Figure 1B–D,F). The CAF-RO group, but not the CAF-R group, also showed intermediate values of rWAT, between CAF and STD (*p* < 0.001; Figure 1A). Moreover, the three cafeteria groups showed increased subcutaneous WAT compared with the STD group (*p* < 0.001; Figure 1E).

**Figure 2:**
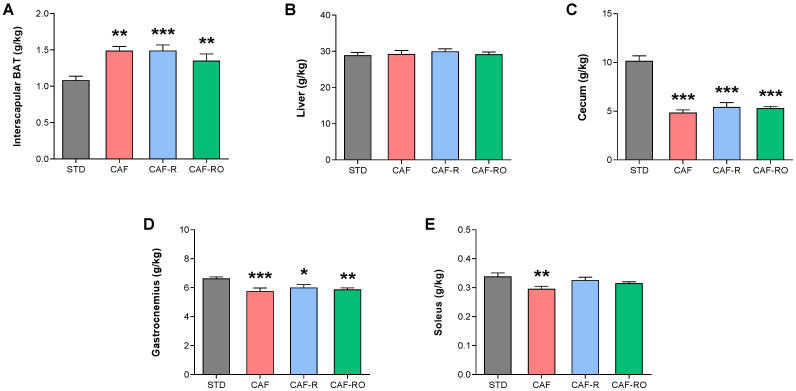
Effects of the dietary treatments on the relative weight of iBAT (**A**), liver (**B**), cecum (**C**) and gastrocnemius (**D**) and soleus (**E**) skeletal muscles. Tissue weights in grams were relativized to total body weight in kg. Data are expressed as the mean ± SEM. * *p* < 0.05, ** *p* < 0.01, *** *p* < 0.001 vs. STD group.

Besides the changes in the WAT depots, the three cafeteria groups displayed a significant increase in iBAT weight compared with the STD group (*p* < 0.001; Figure 2A). These groups also showed decreased cecum weight (*p* < 0.001; Figure 2C), whereas no differences were observed in liver weight (Figure 2B). As for skeletal muscles, the weight of gastrocnemius muscles was diminished in the three cafeteria groups compared with the STD group (*p* < 0.01; Figure 2D), whereas the soleus was only reduced in the CAF group (*p* < 0.05; Figure 2E).

### 3.2. mRNA Expression in HPT

**Figure 3:**
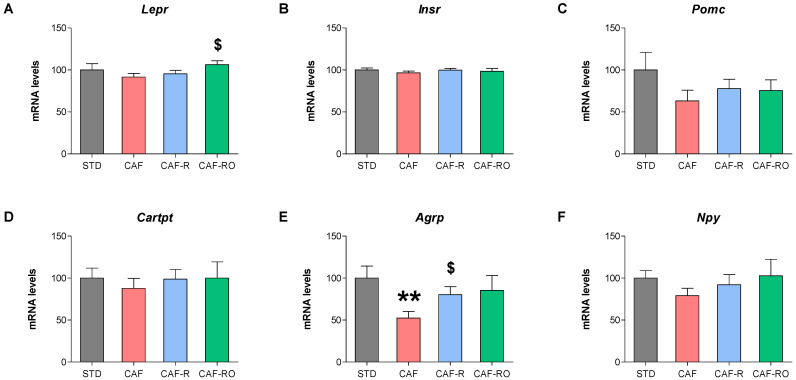
Effects of the dietary treatments on the HPT mRNA levels of *Lepr* (**A**), *Insr* (**B**), *Pomc* (**C**), *Cartpt* (**D**), *Agrp* (**E**) and *Npy* (**F**). *Lepr*, leptin receptor; *Insr*, insulin receptor; *Pomc*, proopiomelanocortin; *Cartpt*, cocaine- and amphetamine-regulated transcript; *Agrp*, agouti-related peptide; *Npy*, neuropeptide Y. Data are expressed as the mean ± SEM and relativized to the STD values (%). ** *p* < 0.01 vs. STD group; $ *p* < 0.05 vs. CAF group.

To analyze the effects of the diets on energy-balance regulation, we analyzed the mRNA levels of several genes involved in energy homeostasis in the HPT. As for the leptin receptor, the results showed that the CAF-RO diet increased its expression levels compared with the CAF diet (*p* < 0.05; Figure 3A). No significant differences were observed in the mRNA levels of the insulin receptor among groups (Figure 3B). As for the target genes of leptin, no differences were observed in the expression levels of *Pomc*, *Cartpt* and *Npy* genes (Figure 3C,D,F). However, the *Agrp* mRNA levels were different depending on the diet, such that they were significantly lower in the CAF group than in the STD (*p* < 0.01), the CAF-R group showed similar values to the STD but higher than the CAF (*p* < 0.01), and the CAF-RO group was not significantly different from any other group (Figure 3E).

### 3.3. mRNA Expression in mWAT

**Figure 4:**
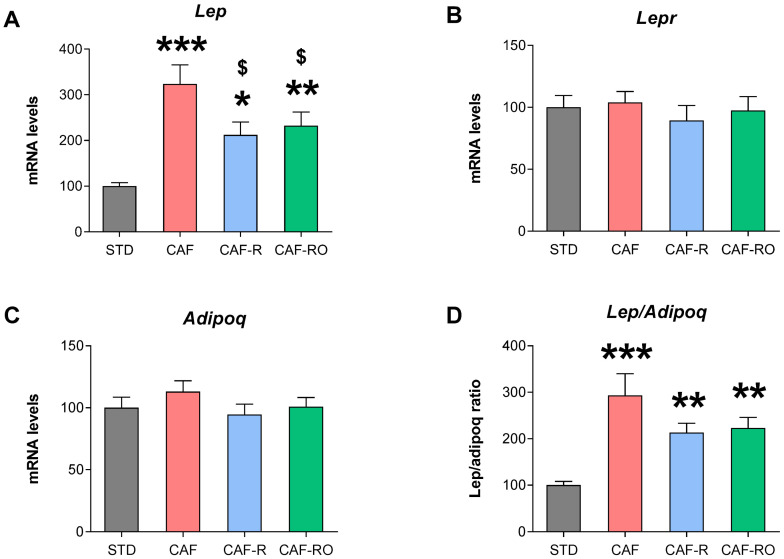
Effects of the dietary treatments on the mRNA levels of *Lep* (**A**), *Lepr* (**B**), *Adipoq* (**C**) and *Lep*/*Adipoq* ratio (**D**) in the mWAT. Data are expressed as the mean ± SEM and relativized to the STD values (%). * *p* < 0.05, ** *p* < 0.01, *** *p* < 0.001 vs. STD group; $ *p* < 0.05 vs. CAF group.

To study whether the diets were modifying adipokine expression in WAT, we analyzed the mRNA levels of *Lep*, *Lepr* and *Adipoq* in the mesenteric fat as a representative of the abdominal adipose tissue depots. The results showed that cafeteria feeding increased the *Lep* mRNA levels, with the CAF group showing the highest expression, the CAF-R and CAF-RO groups intermediate expression, and the STD group the lowest (*p* < 0.001; Figure 4A). While the diets did not induce changes in *Adipoq* mRNA levels, the *Lep*/*Adipoq* ratio was higher in the three cafeteria groups than the STD group (*p* < 0.001; Figure 4D). Finally, no significant differences appeared for the *Lepr* gene.

## 4. Discussion

In the present study we have evaluated the effects of a calorie-restricted cafeteria diet and its supplementation with oleuropein on adiposity, and mRNA expression of target genes involved in energy balance regulation in the hypothalamus and WAT. The dietary interventions started at the age of 12 weeks and were compared with standard chow and cafeteria feeding.

The CAF diet increased the relative weight of both subcutaneous and abdominal WAT depots, and therefore the adiposity index. Furthermore, CAF diet induced a decrease in the gastrocnemius and soleus muscle mass, indicating a sarcopenic condition which, together with the increased and generalized adiposity, characterizes obesity [29]. CAF diet also increased the iBAT mass which would be in line with the low FE observed (see Supplementary Table S1), since loss of usable energy as heat by the diet-induced non-shivering thermogenesis (DIT) process in brown fat is a physiological response to a chronic positive energy balance [30]. All the changes reported here in CAF animals after 22 weeks of CAF feeding (the sum of the first 8-week period and the second 14-week period) concur with those obtained in a previous study where the CAF diet was administered for a shorter period of 8 weeks [26]. Interestingly, there, we showed that despite the greater iBAT mass due to the CAF diet, the mRNA expression of the thermogenic *Ucp1* gene in this tissue was not modified, nor were those of *Pgc1*, *Tfam1* or *Nrf1*, which are involved in mitochondrial biogenesis, also related with energy expenditure mechanisms.

The analysis of the HPT mRNA expression of some of the main leptin and insulin target genes related to the regulation of energy balance showed that CAF diet induced a decrease in the expression of the orexigenic *Agrp* gene, which would depress food intake signals in order to counteract the increased body weight and adiposity [31,32,33]. However, no significant differences in the expression of the other orexigenic gene *Npy* were observed. The CAF diet did not cause an increase in the expression of the anorexigenic *Pomc*/*Cartpt* pathway, either, another expected physiological response to a chronically positive energy balance [34]. All this suggests that the hyperleptinemia and hyperinsulinaemia reported in CAF-fed animals [27] without a complete hormone response at the central nervous system level could indicate a certain degree of resistance to both hormones, a frequent alteration in obesity [35,36]. In fact, we have also reported an increase in the HOMA-IR values as a result of CAF feeding, which is known to be related to the excessive adiposity and relative decrease in muscle mass promoted by this type of diet [27,37,38]. The fact that the hypothalamic expression of the leptin receptor (*Lepr*), regulated by its own hormone ligand, was not altered due to the CAF diet would be in line with this obesity-induced hormone resistance [35,39,40].

We also studied the effects of the obesogenic CAF diet on the mRNA expression of leptin, adiponectin and the leptin receptor in abdominal (mesenteric) WAT. The adipocytes in WAT are the main source of leptin in the body and leptin serum levels are directly related to the amount of this tissue and to triacylglycerides [41]. WAT also secretes adiponectin, an anorexigenic adipokine responsible for, among other functions, the suppression of hepatic glucose production and fatty acid oxidation (lipolysis). Consequently, the leptin/adiponectin ratio (LAR) is considered a measure of whole-body insulin sensitivity and cardiovascular risk in humans [42,43]. Our results showed an increase of more than three times the STD *Lep* mRNA expression levels due to the CAF diet, in accordance with the higher relative weight of mWAT exhibited by the animals fed this diet. On the other hand, no effect was observed on the adiponectin expression levels. However, LAR was increased due to the CAF diet, indicating a balance between these two adipokines in favor of leptin and indicating, again, a hormone resistant state [44,45,46]. Further studies analyzing protein levels of these adipokines and expression of other adipocyte regulatory peptides as well as other body adipose depots may be of interest to better characterize the mechanisms involved in the obesogenic effects of the CAF diet.

Once obesity was induced in adult animals with the CAF diet, we aimed to assess whether a 14-week restricted cafeteria diet, alone or supplemented with the polyphenol OLE, was able to revert or ameliorate the CAF diet-induced alterations described above. The two dietary treatments induced a different eating pattern with lower carbohydrate and higher protein consumption compared to the CAF diet. They also increased FE, although without completely normalizing it, suggesting a partial recovery of the balance between the calories ingested and the body weight gained. These effects of CAF-R diet were also observed in our forementioned previous study where this dietary treatment was administered to young rats from weaning onwards for eight weeks [26]. However, whereas in that study we observed a reduction in iBAT mass compared to the CAF diet, which was accompanied by lower mRNA expression levels of *Ucp1*, *Nrf1* and *Tfam1* in this tissue, here the effect of CAF-R diet on FE was produced without changes in the relative weight of iBAT mass at the end of the study, which remained higher than in the STD group.

OLE supplementation did not exert differential effects on intake and FE parameters with respect to the CAF-R diet alone. Shen et al. [47] reported that OLE administration combined with a high-fat diet (HFD) diminished food intake and reduced the FE ratio in male mice. In contrast, Myoung et al. [48] observed that apigenin supplementation (an OLE constituent) combined with HFD in male mice inhibited food intake at low doses (0.05%) and increased the energy expenditure and FE ratio. Oi-Kano et al. [49,50] reported that OLE administration in HFD-induced obese male rats increased thermogenesis via increasing the *Ucp-1* expression in BAT. Other studies administering polyphenolic compounds to obese animals, such as tyrosol or grape seed proanthocyanidin extract, have also reported an increase in thermogenesis via the activation of *Ucp-1* and energy expenditure [51,52].

Moreover, the CAF-R diet diminished body fat accretion, more specifically the abdominal adiposity, and increased the soleus muscle mass, improving the body composition in obese animals. This effect was more remarkable when the restrictive diet was supplemented with OLE, which caused an additional reduction in the retroperitoneal abdominal fat. Recent studies have reported the protective function of subcutaneous WAT, which is not considered a risk factor for metabolic diseases [53,54]. On the contrary, the accumulation of fat in the abdominal WAT and in other tissues such as the liver, heart and muscles is causally related to insulin resistance, impaired glucose homeostasis and cardiovascular diseases (CVD) [53]. In a similar way, the OLE supplementation of the CAF-R diet also corrected metabolic markers such as serum glucose and insulin sensitivity (see Table S2), in addition to triacylglycerides, insulin, and insulin resistance levels whereas the CAF-R diet alone only normalized the insulinaemia [27]. These results reinforce the potential beneficial effect of the combination of OLE with a restricted cafeteria diet on reverting obesity-induced metabolic dysregulations. Other authors have described similar results when administering OLE as a bioactive compound for obesity treatment. Fki et al. [55] used 10-week-old male Swiss rats that were administered a HFD supplemented with oral OLE (16 mg/kg/day) for 60 days and observed a reduction in the final body weight and in the relative weight of eWAT at the end of the study as well as a decrease of the plasma glucose, insulin, insulin resistance and leptin levels. Similarly, Vezza et al. [15] observed a reduction in the body weight increase and in the weight of eWAT after 5 weeks of a HFD supplemented with OLE (1, 10, 25 mg/kg) in five-week-old male mice, and Kuem et al. [56] reported reduced final body weight and abdominal mass in male mice of the same age that were administered an HFD with 0.03% OLE for 10 weeks.

Regarding the effects of the dietary treatments on hypothalamic mRNA expression of energy balance related genes, the obesogenic CAF diet decreased the *Agrp* mRNA expression as a homeostatic regulatory mechanism, while the CAF-R diet normalized it to similar levels as the STD group. OLE supplementation did not exert additional effects on the expression of this orexigenic gene. These results can be explained by the amelioration of the obesity that CAF-R and CAF-RO diets promoted by decreasing the total and abdominal adiposity, as well as some biometric, food intake and biochemical parameters [27]. Interestingly, OLE supplementation increased hypothalamic *Lepr* expression, which suggests a greater sensitivity to this hormone and the possibility that other leptin target genes may be affected [57]. This result would also be in accordance with the lower insulinaemia and insulin resistance elicited by this polyphenol in combination with the restrictive diet [27]. In Lu et al. [58], rats fed with HFD also exhibited a downregulation of *Agrp* expression levels and green tea polyphenol supplementation restored normal levels of *Agrp*. Several other compounds from vegetal sources have been shown to reach the HPT and improve central leptin sensitivity by increasing the expression of LEPRb, the phosphorylation of STAT3 and the expression of downstream neuropeptides. Thus, apigenin, ginsenoside Rb1, teasaponin, resveratrol, and yerba mate extracts, among others, increased the expression of POMC in the HPT and/or inhibited the orexigenic neuropeptides AGRP and NPY, suggesting their effectiveness for modulating food intake and energy expenditures [48,59,60]. Other dietary supplements such as leucine have also been described to improve leptin sensitivity in rats on HFD [61]. Further analysis of the protein levels of leptin target genes as well as the expression of leptin signaling genes such as JAK2 and STAT3 would help to better elucidate the effects of our dietary treatments on these pathways.

Concerning the effect of the CAF-R diet on the mWAT adipokine mRNA expression, it was also able to reduce the increased leptin mRNA levels compared to the CAF diet, although without reaching the values of the STD group. This treatment did not modify the expression of adiponectin in adipose tissue, although it did partially reduce the LAR without normalizing it. These results are in accordance with the reduction of adiposity, specifically abdominal adiposity, promoted by the CAF-R diet. Moreover, *Lepr* expression was not altered in any of the experimental groups, even in the CAF group where a greater increase of leptin expression was found. This further indicates a leptin resistant state that would also affect adipose tissue, which is sensitive to the action of this hormone, through a negative feedback loop regulatory mechanism (autocrine action) [62]. No additional effects of OLE were observed on these parameters. However, other authors have described changes in adipokine expression due to OLE administration. Van der Stelt 2015 [63] described a reduction of leptin expression in the eWAT of male mice fed a HFD, and Vezza et al. [15] reported increased adiponectin and leptin receptor expression in the eWAT in HFD-induced obese male mice after oral gavage of different OLE doses for five weeks.

In summation, our study reports valuable information about the effects and possible mechanisms of action of oleuropein in combination with the calorie restricted cafeteria diet in obese rats, specifically on the regulation of adiposity and on the mRNA expression of genes involved in hypothalamic anorexigenic and orexigenic pathways as well as the adipokine expression in white adipose tissue. Further studies testing OLE outcomes in female rats to evaluate putative differential sex effects and to translate results to female animals are highly advisable. 

## 5. Conclusions

The administration of the calorie-restricted cafeteria diet ameliorated the obesity-related alterations; specifically, it improved the body composition, reducing adiposity, especially abdominal fat depots, and sarcopenia in male rats. Furthermore, it increased the hypothalamic mRNA expression of the anorexigenic *Agrp* gene and reduced leptin expression in the mWAT. Notably, the OLE supplementation of the CAF-R diet additionally helped to blunt the increase in abdominal adiposity and increased hypothalamic *Lepr* mRNA expression, which suggests sensitization to the action of the leptin hormone. Therefore, our results indicate that the calorie-restricted cafeteria diet could be a useful experimental strategy to constrain obesity and to be combined with bioactive compounds. However, other experiments including female animals and more analyses with different dose ranges and/or longer intervention periods in both sexes would be needed to better determine the additional OLE effects and the mechanisms of its actions in overweight and obesity. 

## Data Availability

The data presented in this study are available in the main article and the supplementary materials.

## Supplementary Materials

The following supporting information can be downloaded at: https://www.mdpi.com/article/10.3390/metabo13020147/s1, Table S1: Feed efficiency and percentage of the energy intake coming from the different macronutrients; Table S2: Serum parameters.
