# Supplementary material for: Effects of a Calorie-Restricted Cafeteria Diet and Oleuropein Supplementation on Adiposity and mRNA Expression of Energy Balance Related Genes in Obese Male Rats
Source: Metabolites. 2023 Jan 18;13(2):147. doi: 10.3390/metabo13020147 (PMC9965300; doi:10.3390/metabo13020147)
Supplement: Supplementary file 1 [file metabolites-13-00147-s001.zip › metabolites-2120972-supplementary.pdf]

Table S1. Feed efficiency and percentage of the energy intake coming from the different macronutrients.

	STD	CAF	CAF-R	CAF-RO
Feed efficiency (%)	2.55 ± 0.08	1.33 ± 0.08***	1.82 ± 0.05***$$$	1.85 ± 0.09***$$$
Energy intake from protein (%)	19.7	10.1 ± 0.2***	12.3 ± 0.2***$$$	12.2 ± 0.2***$$$
Energy intake from carbohydrate (%)	66.2	51.2 ± 0.4***	48.9 ± 0.5***$$$	48.9 ± 0.6***$$$
Energy intake from fat (%)	12.4	38.4 ± 0.5***	38.3 ± 0.7***	38.5 ± 0.8***

Feed efficiency (FE) was calculated as the quotient between the final body weight gain (in grams) and the total energy consumed (in kilocalories) and expressed as a percentage (*100). Daily intake of relative proteins, carbohydrates, and fats are shown as the percentage of the average total daily energy intake (kcal) coming from each macronutrient type. Data are expressed as the mean±SEM. +++p<0.001 vs. STD group (obesity-induction period). ***p<0.001 vs. STD group; $$p$<0.05, $$$p$<0.01, $$$$p$<0.001 vs. CAF group (dietary treatments period).

Table S2. Serum parameters.

	CAF diet-induced obesity period		Dietary treatments period			
	STD	CAF	STD	CAF	CAF-R	CAF-RO
	Week 9		Week 19			
Glucose (mmol/L)	5.59 ± 0.15	6.13 ± 0.08 ++	5.38 ± 0.15	5.96 ± 0.22 *	6.19 ± 0.18 **	5.79 ± 0.31
R-QUICKI	0.354 ± 0.007	0.334 ± 0.004 +	0.349 ± 0.006	0.321 ± 0.009 *	0.327 ± 0.005 *	0.332 ± 0.007

The revised quantitative insulin sensitivity check index (R-QUICKI) was assessed using the formula: 1/[log insulin (μU/mL) + log glucose (mg/dL) + log free fatty acids (mmol/L). Data are expressed as the mean ± SEM. +p<0.05, ++p<0.01 vs. STD group (obesity-induction period); *p<0.05, **p<0.01 vs. STD group (dietary treatments period).
